# Micronized sacchachitin promotes satellite cell proliferation through TAK1-JNK-AP-1 signaling pathway predominantly by TLR2 activation

**DOI:** 10.1186/s13020-020-00381-3

**Published:** 2020-09-22

**Authors:** Meng-Huang Wu, Chuang-Yu Lin, Chun-Yin Hou, Ming-Thau Sheu, Hsi Chang

**Affiliations:** 1 Department of Orthopedics, Taipei Medical University Hospital, No. 252 Wuxing St., Taipei, 11031 Taiwan; 2 Department of Orthopedics, College of Medicine, Taipei Medical University, No. 250 Wuxing St., Taipei, 11031 Taiwan; 3 Department of Clinical Application, Center for iPS Cell Research and Application (CiRA), Kyoto University, 53 Kawahara-cho, Shogoin, Sakyo-ku, Kyoto, 606-8507 Japan; 4 Department of Family Medicine, Taipei City Hospital, Zhongxiao Branch, No. 87 Tongde Rd., Taipei, 115 Taiwan; 5 School of Pharmacy, College of Pharmacy, Taipei Medical University, No. 250 Wuxing St., Taipei, 11031 Taiwan; 6 Department of Pediatrics, School of Medicine, College of Medicine, Taipei Medical University, No. 250 Wuxing St., Taipei, 11031 Taiwan; 7 Department of Pediatrics, Taipei Medical University Hospital, No. 252 Wuxing St., Taipei, 11031 Taiwan

**Keywords:** Sacchachitin, Satellite cells, TAK1-JNK-AP-1 signaling pathway, MAPK signal pathway, Muscle regeneration

## Abstract

**Background:**

*Ganoderma* sp., such as *Ganoderma tsugae* (GT), play an important role in traditional Chinese medicine. *Ganoderma* sp. contains several constituents, including Sacacchin, which has recently drawn attention because it can not only enhance the repair of muscle damage but also strengthen the muscle enforcement. Although *Ganoderma* sp. have a therapeutic effect for neuromuscular disorders, the underlying mechanism remains unclear. This study investigated the effect and underlying molecular mechanism of micronized sacchachitin (mSC) on satellite cells (SCs), which are known as the muscle stem cells.

**Methods:**

The myogenic cells, included SCs (Pax7^+^) were isolated from tibialis anterior muscles of a healthy rat and were cultured in growth media with different mSC concentrations. For the evaluation of SC proliferation, these cultivated cells were immunostained with Pax7 and bromodeoxyuridine assessed simultaneously. The molecular signal pathway was further investigated by using Western blotting and signal pathway inhibitors.

**Results:**

Our data revealed that 200 µg/mL mSC had an optimal capability to significantly enhance the SC proliferation. Furthermore, this enhancement of SC proliferation was verified to be involved with activation of TAK1-JNK-AP-1 signaling pathway through TLR2, whose expression on SC surface was confirmed for the first time here.

**Conclusion:**

Micronized sacchachitin extracted from GT was capable of promoting the proliferation of SC under a correct concentration.

## Background

*Ganoderma tsugae* (GT) is a representative of *Ganoderma* sp., the traditional medicinal mushrooms that comprise of over 80 species. The history of *Ganoderma* sp. could be traced back to more than 2000 years [1], and it has been used for promoting health and extending life in China, Japan, and other East Asian countries. Investigation of this traditional medicinal mushroom by using modern scientific technology revealed that GT contains more than 400 bioactive substances, including sterols, steroids, fatty acids, polysaccharides, triterpenoids, nucleotides, proteins or peptides, and trace elements. Of these, the polysaccharides (PSs) were identified as the major source for biological activity and therapeutic use [2–4]. PSs possess antitumor [5–7], immune-modulatory [6, 8, 9], antioxidant [10–12], and hypoglycemi [13] effects. *Ganoderma*-based drugs have been developed using these pharmaceutical properties. Moreover, many of these products are authorized by State Food and Drug Administration of China for clinical use. In addition to the therapeutic effects mentioned above, enhancement of muscular strength is another well-known effect of *Ganoderma*-based drugs, which have recently drawn attentions. In fact, *Ganoderma*-based drug has already been in clinical use for treating myopathy in China, since 1973 [14]. According to previous studies, this *Ganoderma*-based drug has exhibited effectiveness in improving muscle strength in neuromuscular diseases, including Duchenne muscular dystrophy [15, 16]. Although this *Ganoderma*-based drug has already been clinically applied for more than 30 years, complete understanding and precise mechanism of this drug in improving muscular regeneration and thereby increasing the muscular mass and strength remains unclear.

The skeletal muscle is a large organ, accounting for approximately 40% of the body weight [17]. Homeostasis of the skeletal muscle is mediated by skeletal stem cells, also known as satellite cells (SCs). SCs reside under basal lamina of myofiber [18] and express Pax7, a paired box (PAX) family of transcription factors [19], as its cell marker. SCs are normally under a quiescent state; however, they are activated on muscle damage to proliferate, differentiate, and fuse to form new myofibers, thereby replacing the damaged tissue and restoring its motor function [20, 21]. The processes of muscle regeneration are complex and regulated by multiple signaling pathways. Mitogen-activated protein kinase (MAPK) signaling pathway is closely associated with muscle damage. At least three distinct MAPK pathway targets modulate the muscle regeneration. Activation of extracellular signal-regulated kinase 1/2 (ERK1/2) signaling pathway increases the number of quiescent SCs [22] and c-Jun N-terminal kinase (JNK) signaling promotes the expansion of the activated SC during regenerative myogenesis [23]. By contrast, p38 MAPK activation inhibits self-renewal and promotes differentiation of SC into myoblasts [24–26]. In addition to MAPK signaling pathway, a recent study revealed that the transforming growth factor-β-activated kinase 1 (TAK1), located upstream of MAPK signaling pathway, plays a key role during muscle regeneration. Activation of TAK1 enhances the expression of JNKs and nuclear factor kappa-light-chain-enhancer of activated B cells (NK-κB), which are essential for SC survival and proliferation [27]. Hence, the therapeutic effect of *Ganoderma* sp. in skeletal muscle atrophy possibly involves TAK1 and MAPK signaling pathway activation in SCs.

In this study, the SCs were isolated from the tibialis anterior (TA) muscle of a healthy rat. These isolated SCs were then cultured in growth media containing different concentrations of micronized sacchachitin (mSC), a novel chitin-polysaccharide extracted from the residue of the *Ganoderma* fruiting body, which can induce the proliferation of the corneal epithelium according to our previous reports [2, 28, 29]. In this study, we successfully identified the optimal concentration of mSC for promoting the proliferation of SCs through co-immunostaining of Pax7 and bromodeoxyuridine (BrdU) assay. Secondly, Western blotting and activator protein-1 (AP-1) assay revealed that the enhanced SC proliferation triggered by mSC was mediated by Toll-like receptor 2 (TLR2)-TAK1-JNK signaling pathway, but not through ERK1/2 or p38 MAPK signaling pathways. This study elucidated the beneficial effect of *Ganoderma* sp. on proliferation of SCs and explained the possible therapeutic mechanism of *Ganoderma* sp. for atrophy for the first time. Furthermore, these data revealed the potential of mSC in clinical application for the treatment of skeletal muscle atrophy.

## Materials and methods

All animal handling procedures were approved by the Institutional Animal Care Committee of Taipei Medical University. The animal living conditions were in accordance with the standards of the Guide for the Care and Use of Laboratory Animals (2011), eighth edition, published by National Research Council and the Guidelines of the Animal Research Committee of Taipei Medical University.

### Isolation of myogenic cells from TA muscle

A procedure described in a previous study [30] was used for isolation of myogenic cells. Briefly, Sprague–Dawley rats aged 6–8 weeks and weighing 190–210 g were used in this experiment. After scarification, the TA muscle was excised and homogenized with scissors. The muscle tissue was digested for 30 min at 37 °C by using 0.5% trypsin–EDTA (Thermo Fisher Scientific, Waltham, MA, USA). The sample was collected through centrifugation at 1500×*g* for 5 min and titrated through a fine pipette. An enriched population of myogenic cells was recovered by differential centrifugation (500×*g* for 1 min followed by 1500×*g* for 5 min). The pellet was suspended in Dulbecco’s Modified Eagle Medium (DMEM) containing 5% horse serum (Sigma-Aldrich, St. Louis, MO, USA). The suspension containing myogenic cells was then filtrated though a nylon membrane (200 μm) and plated onto 0.1% gelatin coated petri dish. The isolated cells were cultured in growth medium that comprised DMEM with 5% horse serum and 10% fetal bovine serum and 2 mg/mL, 200 μg/mL and 20 μg/mL of mSC, under 5% CO_2_ at 37 °C. The cell number was counted after 2 days and 8 days of plating for proliferation evaluation until it was confluent for myofiber quantity evaluation.

### GT extraction

Please see Additional file 1 for details.

### Physical analysis and rheological measurement of mSC

Please see Additional file 1 for details.

### Immunohistochemical staining and BrdU assay

The procedure of immunohistochemical analysis was performed as described in a previous study [30, 31]. Briefly, the cultured cells were fixed in 2% paraformaldehyde. Mouse anti-Pax7 (MAB1675; R&D Systems, Minneapolis, MN, USA) at a dilution of 1:50 and anti-Pax7-Cf750 conjugate (Pax7/CF750; 92284, Biotium, Fremont, CA, USA) antibodies were used for Pax7 staining. Rabbit polyclonal anti-BrdU antibody (ab152095; Abcam, Cambridge, UK) was used according to the manufacturer instructions. In addition, rabbit anti-Toll-like receptor 2 (TLR2, ab191458; Abcam, Cambridge, UK) and mouse anti-Toll-like receptor 4 (TLR4, ab22048; Abcam, Cambridge, UK) antibodies were used as primary antibodies. Regarding secondary antibodies, the FITC-labeled anti-mouse IgG, Cy3-labeled anti-rabbit IgG, FITC-labeled anti-rabbit IgG, and Cy3-labeled anti-mouse IgG (715-095-151, 711-165-152, 711-085-152, 715-165-151; Jackson ImmunoResearch Laboratory, West Grove, PA, USA) were used for Pax7, BrdU, TLR2, and TLR4 detection, respectively. Hoechst 33324 (H3570; Invitrogen, Paisley, UK) and DAPI (Biotium 40011, Fremont, CA, USA) were used for nuclear staining. The samples were analyzed through fluorescence microscopy (Olympus, Tokyo, Japan) or on an AS-MDW system (Leica Microsystems, Wetzlar, Germany). Micrographs were obtained using AxioCam (Carl Zeiss Vision, Hallbergmoos, Germany) or the AS-MDW system (Leica Microsystems). The cell counting was performed 48 h after plating. For cell counting analyses, Pax7 positive (Pax7^+^) and Pax7-BrdU double positive (Pax7^+^ BrdU^+^) cells were enumerated per field (× 100) in the culture dish, and the average values and their standard deviations (SDs) were calculated from 10 fields for each culture dish sample. The average ratio of Pax7^+^ BrdU^+^ cells of each group was acquired using the following formula: (Pax7^+^ BrdU^+^)/Pax7^+^/control [30].

### Western blotting

The extracts of primary cultures taken at 36 h or 8 days after plating were subjected to standard procedures. The protein amount was determined using BioRad protein assay. Equal amounts of protein (30 µg) were electrophoresed on 10% acrylamide gel and electrotransferred to polyvinylidene fluoride membrane, followed by immunoblotting with antibodies for myosin heavy chain (MyHC; 1:1000, GTX73432, GeneTex, Taiwan), phospho-TAK1 (1:1000, Cell signaling, #4508), p-p38 (1:1000, Cell signaling, #4511), p-JNK (1:1000, GTX52328, GeneTex, Taiwan), p-ERK (1:1000, Cell signaling, #4370), and p-NF-kB (1:1000, Cell signaling, #3033). Goat anti-actin-HRP (1:5000, Santa Cruz Biotechnology) was used for protein quantification. The membrane was washed and incubated with secondary anti-mouse IgG-HRP (1:3000, sc-2005, Santa Cruz, California, USA), anti-goat IgG-HRP (1:3000, sc-2020, Santa Cruz, California, USA), and anti-rabbit IgG-HRP (1:3000, GTX213110-01, GeneTex, Taiwan). Detection was performed using the T-Pro LumiLong Plus Chemiluminescence Detection Kit (JT96-K004M, T-Pro Biotechnology, Taiwan) and the blots were directly processed on an Amersham Imager 600 (GE, Boston, USA). Densitometry was performed using ImageJ.

### Signaling pathway inhibition

The inhibition of JNK-MAPK, p38-MAPK, and ERK-MAPK signaling pathways was achieved by addition of SP600125 (s5567, Sigma-Aldrich, St. Louis, MO, USA), SB203580 (s8307, Sigma-Aldrich, St. Louis, MO, USA), and U0126 (19-147, Sigma-Aldrich, St. Louis, MO, USA), respectively. TIRAP (TLR2 and TLR4) inhibitor (tlrl-prslps, Invivogen, San Diego, CA, USA) and TLR2 inhibitor (tlrl-oxpl, Invivogen, San Diego, CA, USA) were also used in this experiment. The all dosages of inhibitors were 1 mg/mL.

### AP-1/c-Jun assay

AP-1 ELISA assay was performed using AP-1 Activity Assay Kit (GeneCopoeia Inc. MD. USA) according to the protocol provided by the manufacturer [32]. Nuclear fraction (50 mg) was mixed with the transcription factor–binding buffer supplied by the manufacturer and was then added to each well of the 96-well plate coated with oligo-DNA fragment containing consensus AP-1 binding sequence. After incubation for 1 h at room temperature (RT), the wells were washed with 200 mL of washing buffer supplied by manufacturer for 1 min. After the final wash, 100 mL of diluted anti-AP-1 antibody (1:1000) solution was added to each well except the blank wells, and the plate was incubated for 1 h at RT with gentle rocking. Then, each well was washed two more times with washing buffer and incubated with 100 mL of diluted peroxidase-conjugated secondary antibody (1:1000) at RT for 1 h. After another two washes, each well was treated with chemiluminescence developing solution followed by 30 min incubation at RT with gentle agitation and light shielding. After incubation, 100 mL of the stop solution was added to each well and absorbance was measured at 450 nm wavelength using a spectrophotometric plate reader. Nuclear extract of MCF-7 cells was used as a positive control for this assay.

### Statistical analysis

Data are presented as mean ± standard deviation (SD). Statistical analyses regarding different mSC concentration were conducted using one-way analysis of variance (ANOVA) with Dunnett’s multiple comparisons comparing the mean of each group to the mean of control. One-way ANOVA with Tukey’s multiple comparisons comparing mean of each groups were conducted for evaluation of SC proliferation with inhibitors. The rest of data were analyzed by unpaired t-test. The significance was set at *p *≤ 0.05. Data were analyzed using GraphPad Prism (version 6.0; GraphPad Software, San Diego, CA, USA).

## Results

### Physical and rheological characterizations of mSC

Physical characteristics of *m*SC have been reported by Chen et al. [29]. It was summarized as the following. mSC was measured by particle size analyzer to show an average effective diameter of 2.06 μm with a mid-range polydispersity index (0.459). But when it was observed under SEM, it shows no sign of particles but appearing to be more like the gel matrix (please see Additional file 1). This indicated that those short fibers of mSC was so small in both length and diameter that they could be hydrated to become nanogel when mSC was dispersed in aqueous medium. Rheological characteristics of *m*SC nanogel have also been reported by Chen et al. [29]. Please also see Additional file 1 for details.

### Proliferation of Pax7^+^ SCs isolated from the TA muscle was significantly enhanced by 200 µg/mL mSC

**Fig. 1:**
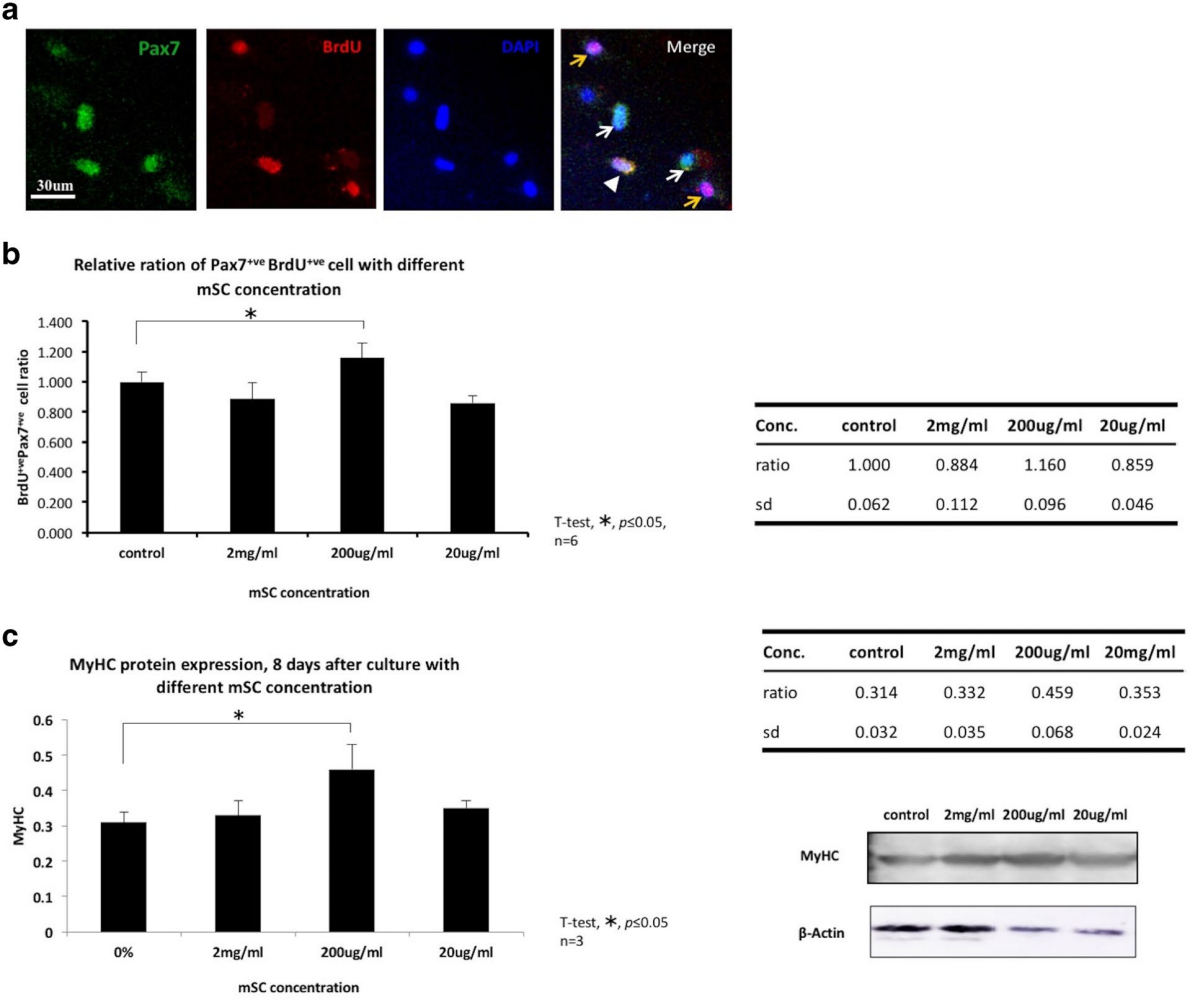
Micronized sacchachitin (mSC) in concentration of 200ug/mL significantly enhanced the proliferation of Pax7 + ve SCs isolated from TA muscle. **a** Co-immunostaining using anti-Pax7 antibody and BrdU assay was performed 2 days after cell plating. Pax7 single positive (Pax7 + ve, white arrow), BrdU single positive (BrdU + ve, yellow arrow) and Pax7-BrdU double positive (Pax7 + ve BrdU + ve, white arrow head) were counted within each group. Scale bar is 30 μm. **b** Micronized sacchachitin (mSC) of 200 μg/ml significantly enhanced the proliferation of Pax7 + ve cells (relative to control, 1.160 ± 0.96, *p < 0.05, n = 6, one-way ANOVA with Dunnett’s multiple comparisons). **c** 8 days after cell plating, MyHC protein within mediums contained different mSC concentration was evaluated through Western blotting assay. Compatible with the results of Pax7 + ve BrdU + ve cell number, mSC of 200 μg/ml showed the highest MyHC (Myosin Heavy Chain) protein expression (0.459 ± 0.068, *p < 0.05, one-way ANOVA with Dunnett’s multiple comparisons)

The concentration of mSC could possibly vary its effect [29]; therefore, we determined its optimal concentration, which could effectively promote SC proliferation. The primary cultured myogenic cells, which were isolated from the TA muscle, were cocultured in growth media that contained different concentrations of mSC, such as 2 mg/mL, 200 µg/mL, and 20 µg/mL. Co-immunostaining using anti-Pax7 antibody and BrdU assay was performed 2 days after cell plating [30]. Pax7 is an SC marker and BrdU assay is commonly used in the detection of proliferating cells; therefore, the number of Pax7^+^BrdU^+^ cells in each assessed mSC concentration could be referred to as actively proliferating SC (Fig. 1a). The results revealed that 200 µg/mL mSC possessed the optimal effectiveness among the three concentrations for promoting Pax7^+^ cell proliferation (200 µg/mL mSC relative to control, 1.160 ± 0.96 and 1.000 ± 0.062 respectively, *p* < 0.01, n = 6, Fig. 1b). This was confirmed by evaluation of MyHC (Myosin Heavy Chain) protein levels in SCs, maintained in different concentrations of mSCs, after 8 days of cell plating through Western blotting. As expected, medium with 200 µg/mL mSC had the highest MyHC protein expression (0.459 ± 0.068 and 0.314 ± 0.032 respectively, *p* < 0.01, n = 3, Fig. 1c); this result was compatible with the Pax7^+^BrdU^+^ coimmunostaining result. Therefore, 200 µg/mL mSC was used for later experiments.

### TLR2 and TLR4 were expressed on SC and the signals of its downstream, TAK1- JNK but not p38, ERK was activated by mSC

**Fig. 2:**
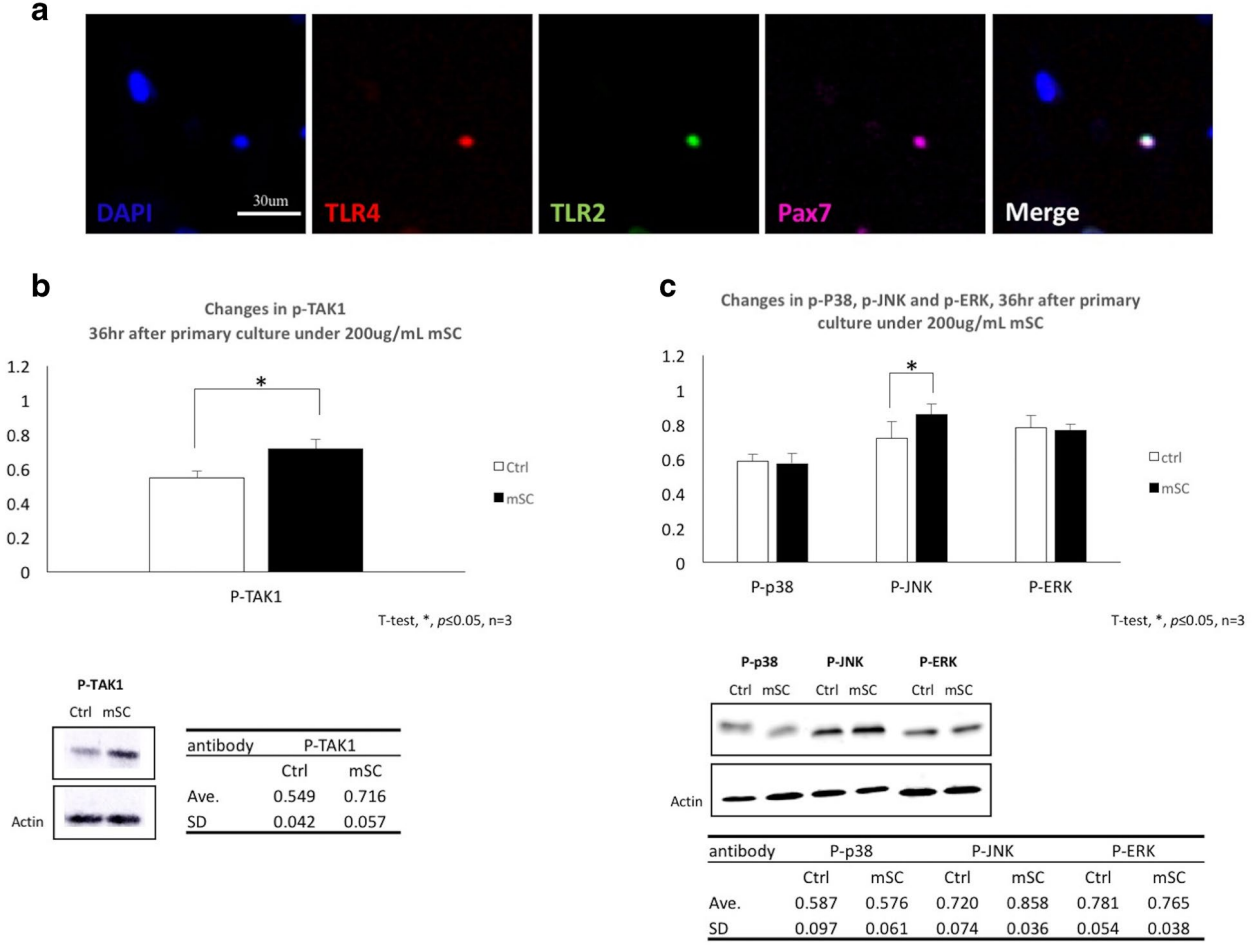
Toll-like receptor 2 and TLR4 were expressed upon SC and the signals of its downstream, TAK1- JNK but not p38, ERK was activated by mSC. **a** The presence of TLR2/4 upon Pax7 + ve SCs were confirmed through immunostaining. Scale bar is 30 μm. **b** Relative to control, a significant elevation of pTAK-1 was admitted under mSC 200 μg/mL (0.716 ± 0.057, *p < 0.05, n = 3, unpaired t-test). **c** Significant elevation was only observed with p-JNK but p-p38 nor p-ERK (0.858 ± 0.036, p < 0.05, 0.576 ± 0.061, p = 0.258, 0.765 ± 0.038, *p *= 0.384, n = 3, unpaired t-test)

Here, 200 µg/mL mSC was the optimal condition to promote SC proliferation, the signaling pathway involved was investigated with this enhancement. According to the previous studies, TLR2 and TLR4 (TLR2/4) express on the surface of myocytes, and their downstream protein kinase MAPK is closely associated with the proliferation of SC [33]. However, the presence of TLR2/4 on SC was not yet confirmed. Therefore, immunostainings were performed to investigate the presence of TLR2/4 on SCs (Pax7^+^) and the results were as expected (Fig. 2a). After confirming the presence of TLR2/4 on SC, the phosphorylation status of TAK1 (p-TAK1) and its downstream molecules, namely p38/MAPK (p-P38), JNK/MAPK (p-JNK), and ERK/MAPK (p-ERK), which form the MAPK signaling pathway, were examined for medium containing 200 µg/mL mSC. As expected, a significant elevation was observed for p-TAK1 (0.716 ± 0.057, *p* < 0.05, n = 3, Fig. 2b). The significant elevation was observed for only p-JNK in MAPK signaling pathway, but not for p-p38 or p-ERK (0.858 ± 0.036, *p* < 0.05; 0.576 ± 0.061, *p* = 0.258; and 0.765 ± 0.038, *p* = 0.384, respectively; n = 3; Fig. 2c).

### The proliferation of SC induced by mSC was effectively suppressed by JNK inhibitor but not by p38 or ERK inhibitor

**Fig. 3:**
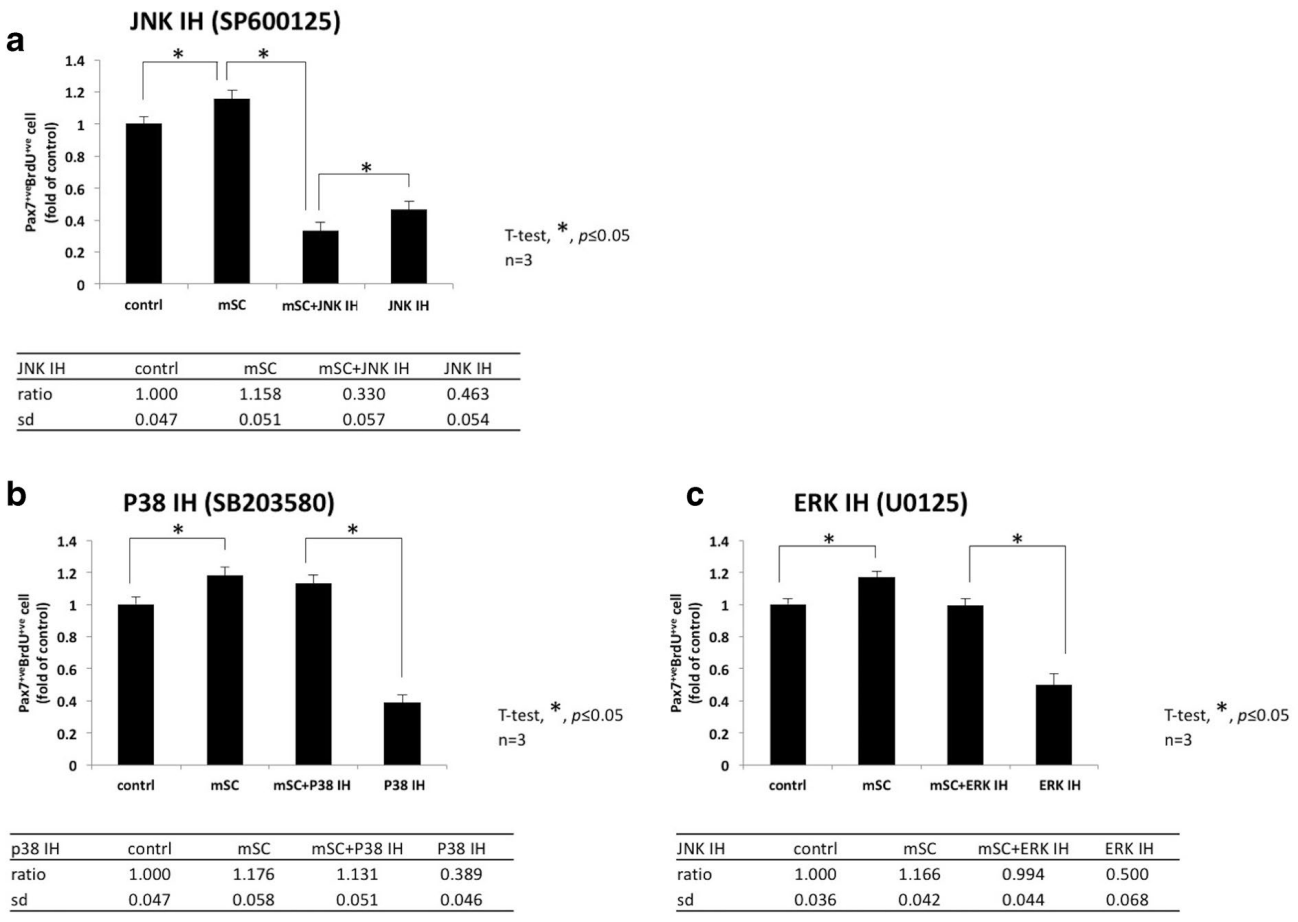
The proliferation of SC induced by mSC could be effectively suppressed by JNK-inhibitor but p38-inhibitor nor ERK-inhibitor. **a** The effective blockage of the enhanced SC proliferation under mSC 200 μg/ml was only observed with JNK inhibitor (mSC + IH relative to JNK IH SP600125, 0.330 ± 0.057 and 0.463 ± 0.054 respectively, *p* = 0.0570, n = 3), **b** but not with p38 inhibitor (mSC + IH relative to p38 IH SB203580, 1.131 ± 0.051 and 0.389 ± 0.046 respectively, ****p < 0.0001, n = 3) nor **c** ERK inhibitor (mSC + IH relative to ERK IH U0126, 0.994 ± 0.044 and 0.500 ± 0.068, ****p < 0.0001, respectively, n = 3, one-way ANOVA with Tukey’s multiple comparisons)

The capability of mSC to activate a specific MAPK signaling pathway was further confirmed by experiments using inhibitors of JNK, p38, and ERK. The effective blockage of the enhanced SC proliferation under the effect of 200 µg/mL mSC was observed with JNK inhibitor (SP600125) only and not with p38 inhibitor (SB203580) nor ERK inhibitor (U0126) (mSC + IH relative to IH, Fig. 3a, JNK inhibitor, 0.330 ± 0.057 and 0.463 ± 0.054, *p* = 0.0570; Fig. 3b, p38 inhibitor, 1.131 ± 0.051 and 1.131 ± 0.051 and 0.389 ± 0.046, *p* < 0.0001; Fig. 3c, ERK inhibitor, 0.994 ± 0.044 and 0.500 ± 0.068, *p* < 0.0001, respectively; n = 3). These results were compatible to the previous results of the phosphorylation status of MAPK signaling pathway molecules, which were induced by mSC. Both results indicated that the JNK/MAPK signaling pathway was the sole pathway to be activated by mSC.

### mSC-induced JNK phosphorylation was predominantly initiated by the activation of TLR2 and mediated by TAK1

**Fig. 4:**
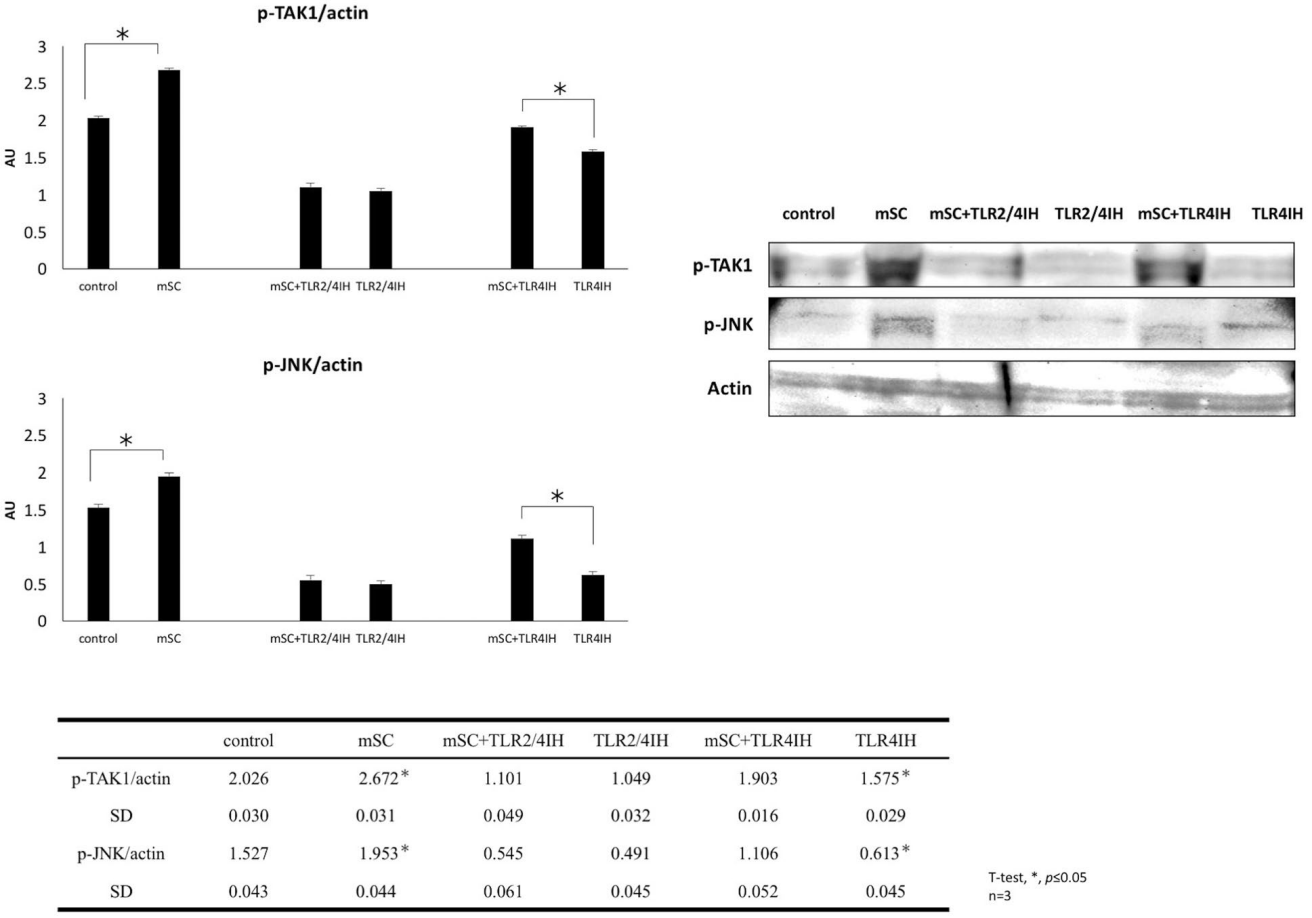
Micronized sacchachitin (mSC) induced JNK/MAPK phosphorylation was predominantly initiated by the activation of TLR2 and mediated by TAK1. The protein level of TAK1 and JNK/MAPK were significantly suppressed by TLR2/4-inhibitor (TLR2/4 IH, 1.10 ± 0.05, p = 0.07, 0.55 ± 0.06, p = 0.13, n = 3, unpaired t-test) but not by TLR4-inhibitor (TLR4IH, 1.903 ± 0.016, 1.106 ± 0.052, *p < 0.05, n = 3, unpaired t-test)

Immunostaining of TLR2 and TLR4 with Pax7 antibody revealed that the TLR2 and/or TLR4 were expressed on SC. Therefore, it was rational to hypothesize that this mSC-induced enhanced SC proliferation was through the activation of TLR2 and/or TLR4. To confirm this hypothesis, the protein levels of p-TAK1 and p-JNK, which were known to be located downstream of TLR2/4, were evaluated under the conditions of mSC-containing medium added with either TLR2/4 inhibitor (TLR2/4 IH) or TLR4 inhibitor (TLR4 IH). The protein levels of p-TAK1 and p-JNK were significantly suppressed by TLR2/4-inhibitor (1.10 ± 0.05, *p* = 0.07 and 0.55 ± 0.06, *p* = 0.13, respectively, n = 3, Fig. 4) and not by TLR4-inhibitor (1.903 ± 0.016 and 1.106 ± 0.052, respectively, *p* < 0.05; n = 3; Fig. 4), although TLR4-inhibitor also exhibited a trend of SC proliferation suppression. These results suggested that the signal transduction induced by mSC was predominantly through the activation of TLR2.

## AP-1 transcriptional activity was significantly enhanced by mSC

**Fig. 5:**
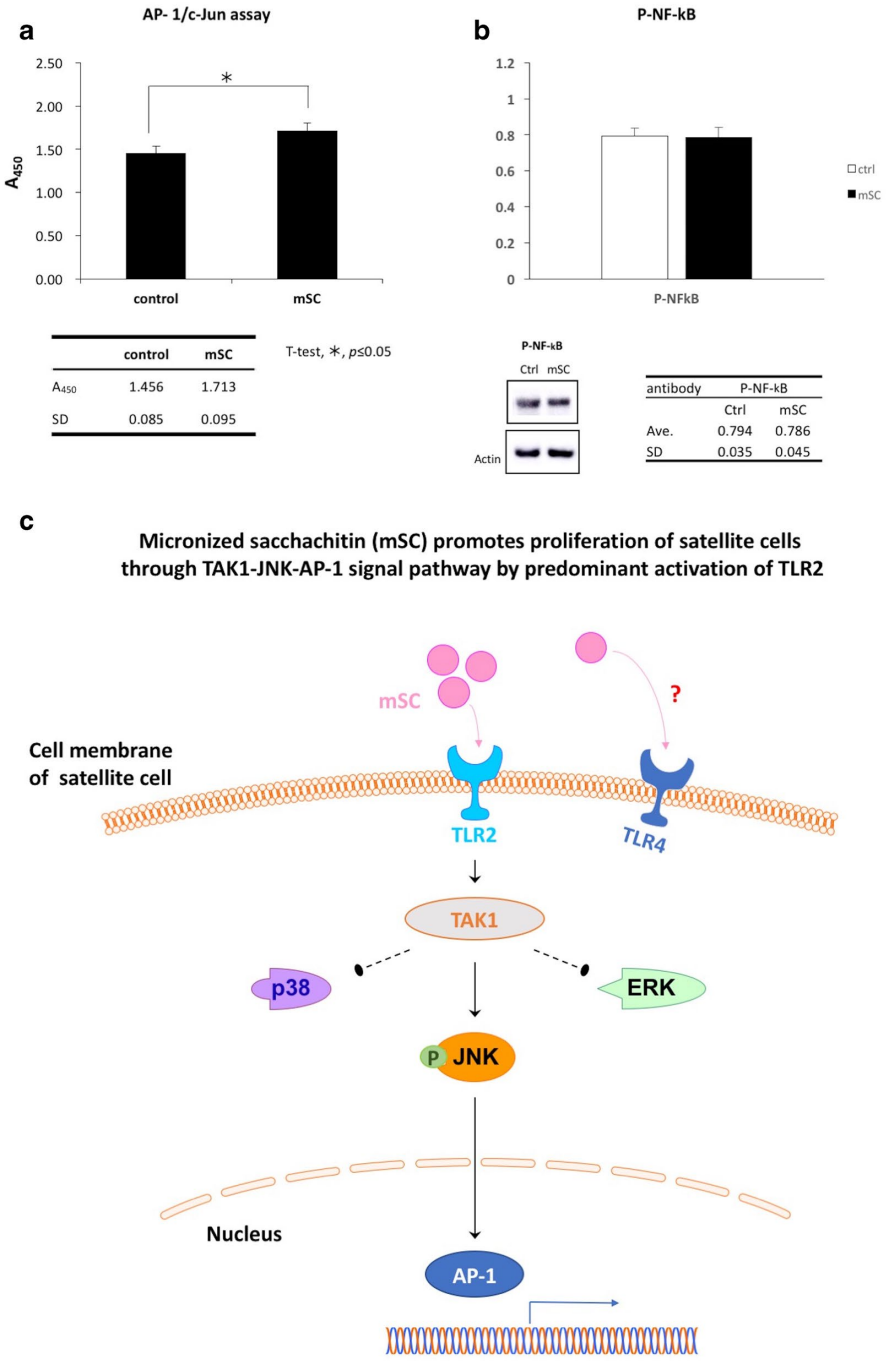
AP-1 transcriptional activity was significantly enhanced by mSC. **a** The AP-1 transcriptional activity of the TA muscle isolated myogenic cells, included Pax7 + ve SCs, which were cultivated with mSC (200 μg/mL) contained medium was significantly enhanced relative to that of the control (1.713 ± 0.095, *p < 0.05, n = 3, unpaired t-test). **b** There was no remarkable increased in phosphorylation of NF-κB admitted (0.786 ± 0.045, p = 0.481, n = 3, unpaired t-test). **c** The enhancement of SC proliferation induced by mSC was predominantly through TLR2 and the activation of TAK 1-JNK-AP-1 signal pathway. Interestingly, JNK was solely activated among the MAPK signaling

Lastly, the changes in AP-1 transcriptional activity of the myogenic cells isolated from TA muscle, included Pax7^+^ population which were cultivated with mSC (200 µg/mL) contained medium was evaluated. The AP-1 transcriptional activity of the myogenic cells in mSC-containing medium was significantly enhanced in relation to that of the control (1.713 ± 0.095, *p* < 0.05, n = 3, Fig. 5a). On the other hand, there was no remarkable increase in phosphorylation of NF-κB (p-NF-kB, 0.786 ± 0.045, *p* = 0.481, n = 3, Fig. 5b).

## Discussion

Skeletal muscular atrophy is an unsolved issue despite of the remarkable progression of modern medication. Although novel treatments, such as photobiomodulation therapy [30], electromyostimulation [34], and amino acid supplementation [35, 36] with or without a combination of traditional rehabilitation, have been proposed to ease the aggravation of atrophy or even to increase the muscle mass, unfortunately the effectiveness of above mentioned therapies are still limited. On the other hand, *Ganoderma* sp., the traditional Chinese medicinal mushrooms, which were popularly used to improve pathological conditions, such as hepatic functional impairment and cancer therapy, have recently drawn attention due to their core component, “sacchachitin” that was clinically found to be capable of improving muscular atrophy. Although this *Ganoderma*-based drug has been applied clinically [14], its pharmacological mechanism remains unclear. As our previous reports revealed [29] that mSC, an original product of our team, can enhance the proliferation of skin cell. However, its effect on myogenic cells, the SCs in particular, has not yet been examined. This study investigated the effect of mSC on SC, which play a critical role during postnatal muscle regeneration, and simultaneously unveil the molecular mechanisms underlying the phenomenon.

In this study, different concentrations of mSC, such as 2 mg/mL, 200 µg/mL, and 20 µg/mL, were added to the SC growth medium. These mSC-containing media were used for culturing of myogenic cells, which were isolated from TA muscle of a healthy rat, revealed presence of Pax7^+^ SCs. Through the coimmunostaining with anti-Pax7 antibody and BrdU cell proliferation assay, we confirmed that 200 µg/mL mSC was the optimal condition to effectively promote the proliferation of SCs (Pax7^+^BrdU^+^). Micronized sacchachitin could boost the proliferation of SC up to 16.1% higher than that of control. Notably, the effect of mSC on SC proliferation appeared as a U-shaped or “biphasic” response; therefore, a risk of adverse effect of mSC on SC proliferation depending on the concentration was speculated. Therefore, the effect of mSC could be a double-edged sword for SC proliferation based on the applied concentration.

Molecular signaling pathways involved in the enhancement of mSC-induced SC proliferation were investigated. TLR2 and TLR4 are expressed on myocytes [37, 38]; however, their presence on SC was not identified. In this study, we confirmed the presence of TLR2 and TLR4 on SC for the first time. Although both TLR2 and TLR4 were expressed on SC, results of our experiments revealed that the mSC-induced enhanced SC proliferation could not be significantly suppressed by TLR4 inhibitor. This result suggested a preferable role of TLR2 in mSC induced SC proliferation. This result was compatible with the previous reports, which revealed a significant role of TLR2 during muscle regeneration, because TLR2 signal could be upregulated by lipopolysaccharides and this upregulation was considered critical for muscle regeneration as this process is important for SC proliferation [39, 40]. Notably, the biphasic effect of mSC on SCs resembled the role of TLR2 signaling pathway, which could be dependent on the property of inflammatory condition that triggered the muscle regeneration, such as acute or chronic muscle injury. The upregulated TLR2 induced proinflammatory environment, which was favorable for muscle regeneration in acute muscle injury; by contrast, a reduced inflammatory environment, facilitated by suppressing TLR2 signaling pathway, is beneficial for chronic muscle injury [40]. Taken together, the optimal mSC concentration can differ based on the characteristics of the muscular atrophy. Thus, a careful evaluation of appropriate mSC concentration, favorable for distinct muscular atrophy type, is important.

TAK1 is located downstream of TLR2 signaling pathway. A recent report revealed that it has an essential role in SC proliferation during muscle regeneration because the inactivation of TAK1 caused precocious differentiation of SC, which eventually led to an inhibition of muscle regeneration [27]. Furthermore, the SC proliferation was also known to be closely involved with MAPK signaling pathway, which is further downstream of TAK1. In this study, the capability of mSC to activate both TAK1 and MAPK signaling pathway was confirmed. However, among the MAPK signaling pathways, JNK signaling pathway was solely activated. This result was also compatible to the previous report, which revealed that the activation of JNK signaling pathway promotes the SC expansion during muscle regeneration [23]. Although either ERK1/2 or p38 MAPK signaling pathways were not activated by mSC, according to our data, the results could not simply be explained as the incapability of mSC for increasing the number of quiescent SC or to boost SC differentiation into myoblast by activating ERK1/2 and p38 MAPK signaling, respectively, because we investigated the optimal mSC concentration by only evaluating the increase in Pax7^+^BrdU^+^ cell number but not the cell counts of quiescent SC (Pax7^+^MyoD^−^) and myoblast (Pax7^+^MyoD^+^).

The activation of AP-1, a transcription factor downstream of TAK1-JNK signaling pathway, was verified to be compatible with previous reports: AP-1 positively regulated the cell proliferation via c-Jun signaling [41]. Since, c-Jun is a downstream transcription factor of JNK, it is reasonable to speculate the effect of mSC, which enhanced the SC proliferation in the present study was through the activation of TAK1-JNK-AP-1 signaling pathway.

AP-1 is closely involved with the regulation of matrix metalloproteinase-9 (MMP-9) [42, 43]; however, the changes in MMP-9 were not evaluated in this study. Therefore, it was difficult to determine the correlation between MMP-9 and mSC. In addition, MMP-9 activity could be promoted by mSC because an elevation of AP-1 activity was observed. These results might contradict the results of our previous study, which revealed that mSC could inhibit the MMP-9 activity, whereas the inhibition of MMP-9 led to an increase in SC proliferation and Notch expression, which is also beneficial for SC proliferation. We suspect that the inconsistency between our current and previous studies may be due to the distinct inflammatory conditions, namely chronic and acute. The myogenic cells used in the present study were collected from the TA muscle of a healthy rat and the evaluation was performed within 36 h after the collection. Therefore, the experiment model of present study tended to be in an acute, rather than chronic, phase of inflammation. By contrast, the corneal damage model or the muscular dystrophy model in our previous study resembled the model of chronic inflammation. The discrimination between the inflammatory phases of the two models might explain the different responses of SC.

In this study, we addressed the signaling pathway of mSC from surface receptor TLR2 to nucleus transcriptional factor AP1 by using co-immunostaining and western blotting. Because of experimental limitation of Pax7^+^ SC, we used immunofluorescence staining instead of flow cytometry to reveal signaling pathways. Although this approach can properly detect the Pax7^+^ cells and other signaling pathway molecules, the advanced approaches is needed to comprehensively study other related signaling pathways, eq. the AKT-mTOR pathway [44] in the future. For further studies, we will apply the reporters, eq. GFP, combined with Pax7 and label other target molecules for reveal the related signaling pathways more precisely.

## Conclusion

In this study, we demonstrated the capability of mSC to promote SC proliferation for the first time. The enhancement was predominant through TLR2, which were expressed on SC, leading to the activation of TAK1-JNK-AP-1 signaling pathway. Notably, JNK was solely activated among the MAPK signaling pathways (Fig. 5c). However, the effect of mSC on SC could be altered based on mSC concentration. Furthermore, the inflammatory phase of the skeletal muscle could also be an involved factor. Thus, applying a “correct” concentration of mSC to the “appropriate” inflammatory environment of injured skeletal muscle could be critical in determining the effectiveness of the medication. This study used SC from healthy rats; therefore, an experiment using human SCs will be our next project. The current results can provide a better understanding of mSC’s effects on muscle regeneration.

## Supplementary information

**Additional file 1.** Ganoderma tsugae (GT) extraction, physical analysis and rheological measurement of mSC.

## Data Availability

All data generated and analyzed during this study are included in this published article and its additional file.

## Abbreviations

GT: *Ganoderma tsugae*
mSC: Micronized sacchachitin
SCs: Satellite cells
PSs: Polysaccharides
PAX: Paired box
MAPK: Mitogen-activated protein kinase
ERK1/2: Extracellular signal-regulated kinase 1/2
JNK: c-Jun N-terminal kinase
TAK1: Transforming growth factor-β-activated kinase 1
TA: Tibialis anterior
mSC: Micronized sacchachitin
BrdU: Bromodeoxyuridine
AP-1: Activator protein-1
DMEM: Dulbecco’s Modified Eagle Medium
SD: Standard deviations
RT: Room temperature

## References

[1] Pang X, Chen Z, Gao X, Liu W, Slavin M, Yao W, Yu LL (2007). Potential of a novel polysaccharide preparation (GLPP) from Anhui-grown *Ganoderma lucidum* in tumor treatment and immunostimulation. J Food Sci.

[2] Su CH, Sun CS, Juan SW, Hu CH, Ke WT, Sheu MT (1997). Fungal mycelia as the source of chitin and polysaccharides and their applications as skin substitutes. Biomaterials.

[3] Chien RC, Yen MT, Tseng YH, Mau JL (2015). Chemical characteristics and anti-proliferation activities of *Ganoderma tsugae* polysaccharides. Carbohydr Polym.

[4] Chen ML, Hsieh CC, Chiang BL, Lin BF (2015). Triterpenoids and polysaccharide fractions of *Ganoderma tsugae* exert different effects on antiallergic activities. Evid Based Complement Alternat Med.

[5] Zjawiony JK (2004). Biologically active compounds from Aphyllophorales (polypore) fungi. J Nat Prod.

[6] Zhu XL, Chen AF, Lin ZB (2007). *Ganoderma lucidum* polysaccharides enhance the function of immunological effector cells in immunosuppressed mice. J Ethnopharmacol.

[7] Wang G, Zhang J, Mizuno T, Zhuang C, Ito H, Mayuzumi H, Okamoto H, Li J (1993). Antitumor active polysaccharides from the Chinese mushroom Songshan lingzhi, the fruiting body of *Ganoderma tsugae*. Biosci Biotechnol Biochem.

[8] Lin ZB, Zhang HN (2004). Anti-tumor and immunoregulatory activities of *Ganoderma lucidum* and its possible mechanisms. Acta Pharmacol Sin.

[9] Gao XX, Fei XF, Wang BX, Zhang J, Gong YJ, Minami M, Nagata T, Ikejima T (2000). Effects of polysaccharides (FI0-b) from mycelium of *Ganoderma tsugae* on proinflammatory cytokine production by THP-1 cells and human PBMC (I). Acta Pharmacol Sin.

[10] Zhong W, Liu N, Xie Y, Zhao Y, Song X, Zhong W (2013). Antioxidant and anti-aging activities of mycelial polysaccharides from *Lepista sordida*. Int J Biol Macromol.

[11] Yang Q, Wang S, Xie Y, Sun J, Wang J (2010). HPLC analysis of *Ganoderma lucidum* polysaccharides and its effect on antioxidant enzymes activity and Bax, Bcl-2 expression. Int J Biol Macromol.

[12] Zhonghui Z, Xiaowei Z, Fang F (2014). *Ganoderma lucidum* polysaccharides supplementation attenuates exercise-induced oxidative stress in skeletal muscle of mice. Saudi J Biol Sci.

[13] Xiao C, Wu QP, Cai W, Tan JB, Yang XB, Zhang JM (2012). Hypoglycemic effects of *Ganoderma lucidum* polysaccharides in type 2 diabetic mice. Arch Pharm Res.

[14] Zeng P, Guo Z, Zeng X, Hao C, Zhang Y, Zhang M, Liu Y, Li H, Li J, Zhang L (2018). Chemical, biochemical, preclinical and clinical studies of *Ganoderma lucidum* polysaccharide as an approved drug for treating myopathy and other diseases in China. J Cell Mol Med.

[15] Wang ZY, Fu HD, Gao LH (1998). Jisheng Injection advent and application of the nervous system diseases. J Youyi Med..

[16] Wu B, Liu ZC, Xu B (2014). Clinical observation on obesity and hyperlipidemia of liver qi stagnation and spleen deficiency pattern in female patients treated with combined therapy of acupuncture and tapping method. Zhongguo Zhen Jiu.

[17] Forbes RM, Cooper AR, Mitchell HH (1953). The composition of the adult human body as determined by chemical analysis. J Biol Chem.

[18] Mauro A (1961). Satellite cell of skeletal muscle fibers. J Biophys Biochem Cytol.

[19] Seale P, Sabourin LA, Girgis-Gabardo A, Mansouri A, Gruss P, Rudnicki MA (2000). Pax7 is required for the specification of myogenic satellite cells. Cell.

[20] Kuang S, Rudnicki MA (2008). The emerging biology of satellite cells and their therapeutic potential. Trends Mol Med.

[21] Yin H, Price F, Rudnicki MA (2013). Satellite cells and the muscle stem cell niche. Physiol Rev.

[22] Abou-Khalil R, Le Grand F, Pallafacchina G, Valable S, Authier FJ, Rudnicki MA, Gherardi RK, Germain S, Chretien F, Sotiropoulos A, Lafuste P, Montarras D, Chazaud B (2009). Autocrine and paracrine angiopoietin 1/Tie-2 signaling promotes muscle satellite cell self-renewal. Cell Stem Cell.

[23] Shi H, Verma M, Zhang L, Dong C, Flavell RA, Bennett AM (2013). Improved regenerative myogenesis and muscular dystrophy in mice lacking Mkp5. J Clin Invest.

[24] Bernet JD, Doles JD, Hall JK, Kelly Tanaka K, Carter TA, Olwin BB (2014). p38 MAPK signaling underlies a cell-autonomous loss of stem cell self-renewal in skeletal muscle of aged mice. Nat Med.

[25] Jones NC, Tyner KJ, Nibarger L, Stanley HM, Cornelison DD, Fedorov YV, Olwin BB (2005). The p38alpha/beta MAPK functions as a molecular switch to activate the quiescent satellite cell. J Cell Biol.

[26] Troy A, Cadwallader AB, Fedorov Y, Tyner K, Tanaka KK, Olwin BB (2012). Coordination of satellite cell activation and self-renewal by Par-complex-dependent asymmetric activation of p38alpha/beta MAPK. Cell Stem Cell.

[27] Ogura Y, Hindi SM, Sato S, Xiong G, Akira S, Kumar A (2015). TAK1 modulates satellite stem cell homeostasis and skeletal muscle repair. Nat Commun.

[28] Su CH, Liu SH, Yu SY, Hsieh YL, Ho HO, Hu CH, Sheu MT (2005). Development of fungal mycelia as a skin substitute: characterization of keratinocyte proliferation and matrix metalloproteinase expression during improvement in the wound-healing process. J Biomed Mater Res A.

[29] Chen RN, Lee LW, Chen LC, Ho HO, Lui SC, Sheu MT, Su CH (2012). Wound-healing effect of micronized sacchachitin (mSC) nanogel on corneal epithelium. Int J Nanomedicine.

[30] Kou YT, Liu HT, Hou CY, Lin CY, Tsai CM, Chang H (2019). A transient protective effect of low-level laser irradiation against disuse-induced atrophy of rats. Lasers Med Sci.

[31] Chang H, Yoshimoto M, Umeda K, Iwasa T, Mizuno Y, Fukada S, Yamamoto H, Motohashi N, Miyagoe-Suzuki Y, Takeda S, Heike T, Nakahata T (2009). Generation of transplantable, functional satellite-like cells from mouse embryonic stem cells. FASEB J.

[32] Bid HK, Phelps DA, Xaio L, Guttridge DC, Lin J, London C, Baker LH, Mo X, Houghton PJ (2016). The bromodomain BET inhibitor JQ1 suppresses tumor angiogenesis in models of childhood sarcoma. Mol Cancer Ther.

[33] Zhang W, Liu HT (2002). MAPK signal pathways in the regulation of cell proliferation in mammalian cells. Cell Res.

[34] Adams V (2018). Electromyostimulation to fight atrophy and to build muscle: facts and numbers. J Cachexia Sarcopenia Muscle.

[35] Valenzuela PL, Morales JS, Emanuele E, Pareja-Galeano H, Lucia A. Supplements with purported effects on muscle mass and strength. Eur J Nutr. 2019. 10.1007/s00394-018-1882-z 30604177

[36] Dreyer HC, Owen EC, Strycker LA, Smolkowski K, Muyskens JB, Kirkpatrick TK, Christie AD, Kuehl KS, Lantz BA, Shah SN, Mohler CG, Jewett BA (2018). Essential amino acid supplementation mitigates muscle atrophy after total knee arthroplasty: a randomized, double-blind, placebo-controlled trial. JBJS Open Access..

[37] Francaux M (2009). Toll-like receptor signalling induced by endurance exercise. Appl Physiol Nutr Metab.

[38] Zbinden-Foncea H, Raymackers JM, Deldicque L, Renard P, Francaux M (2012). TLR2 and TLR4 activate p38 MAPK and JNK during endurance exercise in skeletal muscle. Med Sci Sports Exerc.

[39] Xu J, Benabou K, Cui X, Madia M, Tzeng E, Billiar T, Watkins S, Sachdev U (2015). TLR4 deters perfusion recovery and upregulates toll-like receptor 2 (TLR2) in ischemic skeletal muscle and endothelial cells. Mol Med.

[40] Hindi SM, Kumar A (2016). Toll-like receptor signalling in regenerative myogenesis: friend and foe. J Pathol.

[41] Shaulian E, Karin M (2001). AP-1 in cell proliferation and survival. Oncogene.

[42] Wang T, Jin X, Liao Y, Sun Q, Luo C, Wang G, Zhao F, Jin Y (2018). Association of NF-kappaB and AP-1 with MMP-9 overexpression in 2-chloroethanol exposed rat astrocytes. Cells.

[43] Tombulturk FK, Soydas T, Sarac EY, Tuncdemir M, Coskunpinar E, Polat E, Sirekbasan S, Kanigur-Sultuybek G (2019). Regulation of MMP 2 and MMP 9 expressions modulated by AP-1 (c-jun) in wound healing: improving role of *Lucilia sericata* in diabetic rats. Acta Diabetol.

[44] Schiaffino S, Dyar KA, Ciciliot S, Blaauw B, Sandri M (2013). Mechanisms regulating skeletal muscle growth and atrophy. FEBS J.

