# Correction to: Micronized sacchachitin promotes satellite cell proliferation through TAK1-JNK-AP-1 signaling pathway predominantly by TLR2 activation

**DOI:** 10.1186/s13020-020-00420-z

**Published:** 2021-02-04

**Authors:** Meng–Huang Wu, Chuang–Yu Lin, Chun–Yin Hou, Ming–Thau Sheu, Hsi Chang

**Affiliations:** 1 Department of Orthopedics, Taipei Medical University Hospital, No. 252 Wuxing St., Taipei, 11031 Taiwan; 2 Department of Orthopedics, College of Medicine, Taipei Medical University, No. 250 Wuxing St., Taipei, 11031 Taiwan; 3 Department of Clinical Application, Center for IPS Cell Research and Application (CiRA), Kyoto University, 53 Kawahara-cho, Shogoin, Sakyo-Ku, Kyoto 606-8507 Japan; 4 Department of Family Medicine, Taipei City Hospital, Zhongxiao Branch, No. 87 Tongde Rd., Taipei, 115 Taiwan; 5 School of Pharmacy, College of Pharmacy, Taipei Medical University, No. 250 Wuxing St., Taipei, 11031 Taiwan; 6 Department of Pediatrics, School of Medicine, College of Medicine, Taipei Medical University, No. 250 Wuxing St., Taipei, 11031 Taiwan; 7 Department of Pediatrics, Taipei Medical University Hospital, No. 252 Wuxing St., Taipei, 11031 Taiwan

## Correction to: Chin Med (2020) 15:100 10.1186/s13020-020-00381-3

Following publication of the original article [1], the authors would like to correct the content under Funding.

The content currently reads:

This study funded by Taipei Medical University Hospital (108TMU-TMUH-16).

The content should be changed to:

This study funded by Taipei Medical University Hospital (109TMU-TMUH-10).

The original article has been updated.
