# Supplementary material for: Micronized sacchachitin promotes satellite cell proliferation through TAK1-JNK-AP-1 signaling pathway predominantly by TLR2 activation
Source: Chin Med. 2020 Sep 22;15:100. doi: 10.1186/s13020-020-00381-3 (PMC7510329; doi:10.1186/s13020-020-00381-3)
Supplement: Supplementary file 1 — Additional file 1. Ganoderma tsugae (GT) extraction, physical analysis and rheological measurement of mSC. [file 13020_2020_381_MOESM1_ESM.docx]

***Additional file***

**Micronized sacchachitin promotes satellite cell proliferation through TAK1-JNK-AP-1** **signaling pathway predominantly by TLR2 activation**

Meng-Huang Wu^1,2^,

Chuang-Yu Lin^3^

Chun-Yin Hou^4^

Ming-Thau Sheu^5,*^

Hsi Chang MD, PhD^6,7,*^

^1^Department of Orthopedics, Taipei Medical University Hospital, No.252 Wuxing St., Taipei 11031, Taiwan;

^2^Department of Orthopedics, College of Medicine, Taipei Medical University, No.250 Wuxing St., Taipei 11031, Taiwan;

^3^Department of Clinical Application, Center for iPS Cell Research and Application (CiRA), Kyoto University, 53 Kawahara-cho, Shogoin, Sakyo-ku, Kyoto 606-8507, Japan; ^4^Department of Family Medicine, Taipei City Hospital, Zhongxiao Branch, No.87 Tongde Rd.,Taipei 115, Taiwan;

^5^School of Pharmacy, College of Pharmacy, Taipei Medical University, No.250 Wuxing St., Taipei 11031, Taiwan;

^6^Department of Pediatrics, School of Medicine, College of Medicine, Taipei Medical University, No.250 Wuxing St., Taipei 11031, Taiwan;

^7^Department of Pediatrics, Taipei Medical University Hospital, No.252 Wuxing St., Taipei 11031, Taiwan

*The authors contributed equally to this study.

Correspondence: Hsi Chang; Ming-Thau Sheu

No. 252 Wuxing St., Taipei 11031, Taiwan; No.250 Wuxing St., Taipei 11031, Taiwan
Telephone: 886-2-27372181 ext. 3715; 886-2-27361661 ext. 6112

Fax: 886-2-27360399; 886-2-23771942

Email: [jamesc@h.tmu.edu.tw](mailto:jamesc@h.tmu.edu.tw); mingsheu@tmu.edu.tw

**Materials and methods**

***GT extraction***

Sacchachitin was prepared from the residue of the fruiting body of GT, which was obtained by solid state cultivation. A method reported in a previous study was used for sample preparation [28, 29]. In brief, 1,000 g of the residue was pulverized and extracted with ethanol for 48 h. The residue was collected and blow-dried at 40°C. The dried mixture was then digested with 1N NaOH at 85°C for 24 h. The residue was collected and washed with deionized water to remove any residual NaOH. H_2_O_2_ (1:3 to weight of the residue) was then used for pigmentation followed by repeated washing with deionized water for removal of any residual H_2_O_2_. The pulp-like residue was then sieved to obtain fibers of 10–50 µm length. Sacchachitin so obtained was micronized into fine powder by the bead-milling method using NANO-M2 model (ITRI; Hsinchu, Taiwan). The specifications of grinding media were φ 0.1 mm YTZ, pin type, and 18 m/s rotor speed. The nanogel of mSC was prepared by adding mSC powder to the sterilized phosphate-buffered saline before application in the study. Characterization of mSC was done as described in a previous study using size-exclusion and thin-layer chromatography.

***Physical analysis and rheological measurement of mSC***

Physical characteristics of *m*SC, including the length of mSC fibers expressed as effective diameter and Zeta potential were measured using a Brookhaven 90Plus Particle Size Analyzer (Brookhaven Instruments Corp, Holtsville, NY) followed the same procedure as reported by Chen et al [29]. Rheological properties of *m*SC nanogel, including linear viscoelastic region, steady viscosity (η) as a function of shear rates, elastic modulus G′ (storage modulus), viscous modulus G′ (loss modulus), and complex vis­cosity (η*), were characterized with HAAKE RheoStress 1 (Thermo Fisher Scientific Inc, Rockford, IL) equipped with a Cone Ø60 mm, 1° _angle (C60/1) followed the same method and procedure reported by Chen et al [29].

***Results***

***Rheological characterizations of mSC***

Rheological characteristics of *m*SC nanogel have also been reported by Chen et al [29]. It was summarized as the following. The viscosities of nanogel dispersions decrease rapidly with an increase of shear rates. this demonstrates shear thinning as a result of elongating the random coil of those mSC fibers interacting with water along the shear direction and breaking down of the network formed by interactions among mSC fibers, which is similar to typical pseudoplastic fluids. In dynamic stress sweep study, constant G′ and G′′ values at the lower shear stress region were observed, while a rapid drop of both values at higher shear stress appeared with the extent of reduction being greater for G′ than that for G′′. This indicated that the network composed of the particle-like domain of mSC fiber-mSC fiber interaction is easier to be broken down than that of the viscous gel domain composed of hydrated mSC fibers. The complex viscosities (η*) of mSC nanogel were observed to decrease with increasing stress after the gel structure of mSC breaks down, at which point the applied shear stress was higher than the yield stress. Overall results revealed that mSC nanogel to be a pseudoplastic flow with lower viscosities at room temperature. The morphological images are also showed in Supplementary figure 1.

As dynamic viscoelasticity test revealed that the G′ value is higher than the G′′ value over the whole range of frequencies at these two stresses, indicating that mSC nanogel is predomi­nantly elastically solid at room temperature. Both G′ and G′′ increase rapidly in the initial period of the frequency increase, suggesting the strong frequency dependence of both modulus. Thereafter, both modules increase slowly as the frequency exceeds 10 Hz, which is likely a result of the formation of a transient 3D percolation network induced by the fast collid­ing rate of mSC fibers at high frequencies. Further, the dynamic temperature ramp curves measured to illustrate that with an increase of temperature, the rheological parameters (G′, G′′, and η*) first gradually increase in the temperature range of 20°C–35°C and then increase remarkably at the temperature around 37°C. These phenomena were explained as a result from the hydrophilicity of mSC fibers, which might have a strong interaction of mSC fiber-mSC fiber and mSC fiber-water via hydrogen bonding.

**
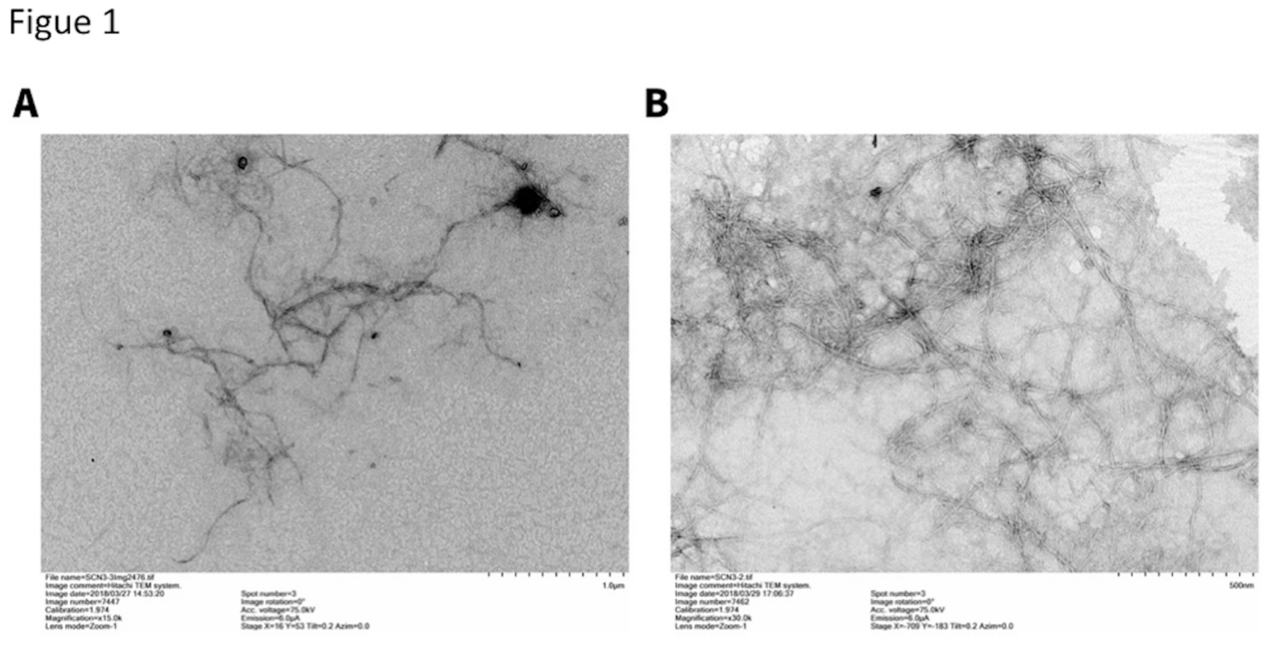
**

**Additional figure S1.** Transmission electron microscopy photographs for micronized sacchachitin at two magnifications of (**A**) x15k and (**B**) x30k.
